# Benefits of adaptive cognitive training on cognitive abilities in women treated for primary breast cancer: Findings from a 1‐year randomised control trial intervention

**DOI:** 10.1002/pon.6232

**Published:** 2023-10-26

**Authors:** Bethany Chapman, Courtney C. Louis, Jason Moser, Elizabeth A. Grunfeld, Nazanin Derakshan

**Affiliations:** ^1^ School of Psychology and Clinical Language Sciences University of Reading Reading UK; ^2^ Department of Psychological Sciences Birkbeck, University of London London UK; ^3^ Department of Psychology Michigan State University East Lansing Michigan USA

**Keywords:** adaptive cognitive training, breast cancer, cancer, cognitive impairment, Oncology, P3, working memory capacity

## Abstract

**Objective:**

While adaptive cognitive training is beneficial for women with a breast cancer diagnosis, transfer effects of training benefits on perceived and objective measures of cognition are not substantiated. We investigated the transfer effects of online adaptive cognitive training (dual *n*‐back training) on subjective and objective cognitive markers in a longitudinal design.

**Methods:**

Women with a primary diagnosis of breast cancer completed 12 sessions of adaptive cognitive training or active control training over 2 weeks. Objective assessments of working memory capacity (WMC), as well as performance on a response inhibition task, were taken while electrophysiological measures were recorded. Self‐reported measures of cognitive and emotional health were collected pre‐training, post‐training, 6‐month, and at 1‐year follow‐up times.

**Results:**

Adaptive cognitive training resulted in greater WMC on the Change Detection Task and improved cognitive efficiency on the Flanker task together with improvements in perceived cognitive ability and depression at 1‐year post‐training.

**Conclusions:**

Adaptive cognitive training can improve cognitive abilities with implications for long‐term cognitive health in survivorship.

## 1 BACKGROUND

Women diagnosed with breast cancer are at a greater risk of developing a series of short‐ and long‐term sequelae including, cancer‐related cognitive impairment (CRCI)^1^ and emotional distress (anxiety and depression).^2^ These adversely impact their survivorship^3^ and quality of life (QoL)^4^ including their workability.^5^, ^6^

Cancer‐related cognitive impairment describes deficits in cognitive functions supported by working memory, such as attention and memory, concentration, task switching, and distractibility, and is one of the most common longer‐term symptoms experienced by women diagnosed with breast cancer.^1^, ^7^ While the impact of CRCI on performance outcomes is mixed^8^ increasing evidence points to the relevance of neurocognitive markers explaining variation in performance outcomes.^9^ Thus, obtaining a multitude of outcome measures spanning behavioural, neural, and self‐report indices of cognitive abilities provides a methodologically comprehensive approach to the study of CRCI on performance.^10^ There is evidence that adaptive cognitive training can improve CRCI, as well as workability in women treated for breast cancer.^11^, ^12^

Women treated for breast cancer have shown abnormalities in electrophysiological markers of cognitive function such as the P3 component of attention,^13^ which indicates the allocation of cognitive resources to task demands (e.g., as measured in a Flanker task measuring response inhibition).^14^ P3 differences in breast cancer patients have been suggestive of reduced sustained attention and poorer allocation of attention to task‐relevant information.^15^ The error‐related negativity (ERN) and error positivity (Pe) are also electrophysiological markers of interest and studied in reaction time conflict tasks, reflecting adjustment of response strategies.^16^

The current study investigates the impact of adaptive cognitive training intervention using adaptive dual *n*‐back training on behavioural and neural indices of cognitive performance and working memory capacity (WMC) in women affected by primary breast cancer. The efficacy of the adaptive dual *n*‐back training intervention on improving cognitive and emotional health has been substantiated in a variety of populations including anxious, depressed, high worriers,^17^ as well as women affected by breast cancer^18^, ^19^ where sustainable reductions in anxiety and perseverative thinking were found.^19^ In a recent qualitative study adaptive dual *n*‐back training improved workability and coping strategies in the workplace, in women with a breast cancer diagnosis.^20^ Most cancer survivors seek support for their CRCI, and cognitive training (72%) is preferred over psychological support (48%) and physical activity (32%).^21^, ^22^ Collectively, these findings highlight the important implications that adaptive cognitive training may have for clinicians and oncologists when considering patient referrals for therapy. Consequently, patients will be better placed to obtain the maximum outcome benefits at the right time.

We assessed transfer‐related gains of adaptive cognitive training on multiple indices of cognitive performance (WMC and inhibitory control) and electrophysiological measures (P3, ERN, and Pe) on cognitive tasks unrelated to the training intervention. We predicted that the intervention group compared with the active control group will show improvements on cognitive and neural indices of performance. We also measured cognitive ability, workability, and emotional health using questionnaires.

## 2 METHODS

Ethical approval was obtained (reference: 181,935) and the study was registered with the International Standard Registered Clinical/soCial sTudy Number (ISRCTN; ISRCTN11333136).

### 2.1 Participants

Women with primary breast cancer (*N* = 80) were recruited via advertisements in support networks via social media between first of February 2019 and 29^th^ of February 2020. Women were screened against the following inclusion criteria: (1) aged 18–65, (2) diagnosis of primary breast cancer, (3) 6–60 months post‐active treatment (chemotherapy and/or radiotherapy), (4) can be receiving hormone blockers, replacement therapies or Herceptin, (5) employed or self‐employed and (6) experiencing a decline in cognitive abilities.

### 2.2 Materials and stimuli

#### 2.2.1 General demographics questionnaire

The General demographics questionnaire (GDQ) (developed by the authors) comprising of 30 questions was used to collect information relating to sociodemographic factors, lifestyle, clinical history, psychiatric history, and current employment.

### 2.3 Primary outcomes and measures

#### 2.3.1 Change detection task

The shortened version of the Change detection task (CDT)^23^ measured WMC. There was a practice session (12 trials, 4 per condition) and 192 experimental trials split into four blocks of 48 trials. Participants started the experimental trials once they had reached ≥50% accuracy in the practice session.

#### 2.3.2 Automated operation span task

The automated OSpan^24^ task was also used to measure working memory. After practice trials, participants completed three blocks of 15 experimental trials (75 letters and 75 maths equations).

#### 2.3.3 Questionnaires

##### Functional assessment of cancer therapy‐cognitive scale—perceived cognitive ability subscale (Version 3)

Perceived cognitive function was measured using the FACT‐Cog‐PCA scale^25^ comprising nine items with a five‐point Likert scale from 0 to 4. The choice of using Perceived Cognitive Ability (PCA) was based on suggestions by Lai et al^26^ that positively framed PCA items may reduce the effects of negative emotional state (such as depression) on cognitive function. Scores range between 0 and 36. Higher scores reflect better perceived cognitive abilities. Cronbach's *α* 0.85.

##### Rumination response scale

Rumination was assessed using the RRS^27^ a 22‐item with a four‐point Likert scale ranging from 1 (‘never’) to 4 (‘almost always’). The total score ranges from 22 to 88. Higher scores indicate higher rumination. Cronbach's *α* 0 0.94.

### 2.4 Secondary outcomes and measures

#### 2.4.1 Work limitations questionnaire

Workability was assessed by the WLQ^28^ comprising 25 items measured on a five‐point Likert scale, with reverse scoring for some subscales. After applying the Work limitations questionnaire (WLQ) formula, scores range from 0 to 100. Higher scores indicate greater workplace difficulty. The mental/interpersonal demands scale was the variable of interest. Cronbach's *α* 0.88.

#### 2.4.2 Hospital anxiety and depression scale—Anxiety scale

Anxiety was assessed using the HADS‐A^29^, a seven‐item inventory with a four‐point Likert scale ranging from 0 to 3. Total score ranges from 0 to 21. Higher scores indicate higher anxiety symptomatology. Cronbach's *α* 0.84.

#### 2.4.3 Centre for epidemiologic studies depression scale

Depression was measured by the CES‐D^30^, a 20‐item scale with a four‐point Likert scale ranging from 0 to 3. Total score ranges from 0 to 60. Higher scores indicate higher depressive symptomatology. Cronbach's *α* 0.92.

#### 2.4.4 European organization for research and treatment of cancer quality of life—Global health status

Quality of life was measured using the EORTC‐QLQ‐C30‐GHS^31^ comprising 2 items measured on a seven‐point scale ranging from 1 (‘very poor’) to 7 (‘excellent’), with scores ranging from 0 to 100. Higher scores reflect better QoL. Cronbach's *α* 0.85.

#### 2.4.5 Electroencephalography

Electroencephalography (EEG) activity was recorded continuously using BrainVision Recorder (Brain Products, Gilching, Germany) from 32, Ag‐AgCl passive electrodes embedded in a standard BrainVision BrainCap (EasyCap) including, both left and right mastoids (TP9 and TP10) during a modified Flanker task. Split‐half reliability was assessed using Spearman‐Brown‐corrected Pearson correlation coefficients between odd and even trials (*SB* = 2*r*_*xy*_/(1 + *r*_*xy*_)). The respective time windows for the P3, ERN, and Pe were determined by visual inspection of the grand average waveforms.

##### Posterior P3

The P3 was defined as the mean activity occurring 300–500 ms after incongruent and congruent stimulus onset on correct trials, at the Pz electrode, where the P3 was maximal.

##### Error‐related negativity

The ERN was defined as the mean amplitude in the post‐response time window from 0 to 100 ms, at the Cz electrode, where ERN was maximal.

##### Error positivity

The Pe was defined as the mean activity occurring in 2 sequential post‐response time windows from 150 to 550 ms where the amplitude was maximal.

#### 2.4.6 Modified standard letter Flanker

The modified standard letter Flanker^32^ measured inhibitory control whilst participants underwent EEG testing. Participants were asked to respond rapidly and accurately using the computer mouse to identify the central letter (target letter) shown within a string of five letters (i.e., incongruent: MM**N**MM or congruent: MM**M**MM). There were 24 practice trials and 480 experimental trials divided into 12 blocks of 40 trials with a 50% congruency rate. Participants' reaction times and response accuracy were calculated, as well as post‐error slowing (difference between response reaction times on correct trials following an error or correct response (EC–CC) and used in the analysis to indicate improved inhibition. Corrections were applied for switching block failure (>= 60% errors).

### 2.5 Dual *n*‐back training (intervention: Adaptive cognitive training) and dual 1‐back training (active control)

Standard versions of dual *n*‐back training and dual 1‐back training [replicated from^18^, ^19^] were utilised (see supplementary material III. Figure 2). During each trial, a single green square appeared in one of eight positions on the grid accompanied by a single letter consonant (*h*, *l*, *c*, *q*, *s*, *r*, *k*, and *t*) spoken by a female voice. Participants were asked to simultaneously remember the location of the green square and spoken consonant. Responses were made via the keyboard when either a single stimulus or both stimuli matched what was presented ‘*n*’ number of trials beforehand. No response was required for a non‐match. Speed and accuracy were emphasised.

An accuracy score of ≥95% increased the difficulty level of ‘*n*’ by one, accuracy of <75% decreased ‘*n*’ by one, and when accuracy was maintained between 75% and 95%, level of ‘*n*’ remained the same on the next block. Participants were required to complete 20 blocks of 20 + *n* trials. Dual 4‐back was the highest achievable level.

Dual 1‐back training (active control) difficulty remained unchanged at 1‐back for all 20 blocks of trials.

### 2.6 Procedure

**FIGURE 1:**
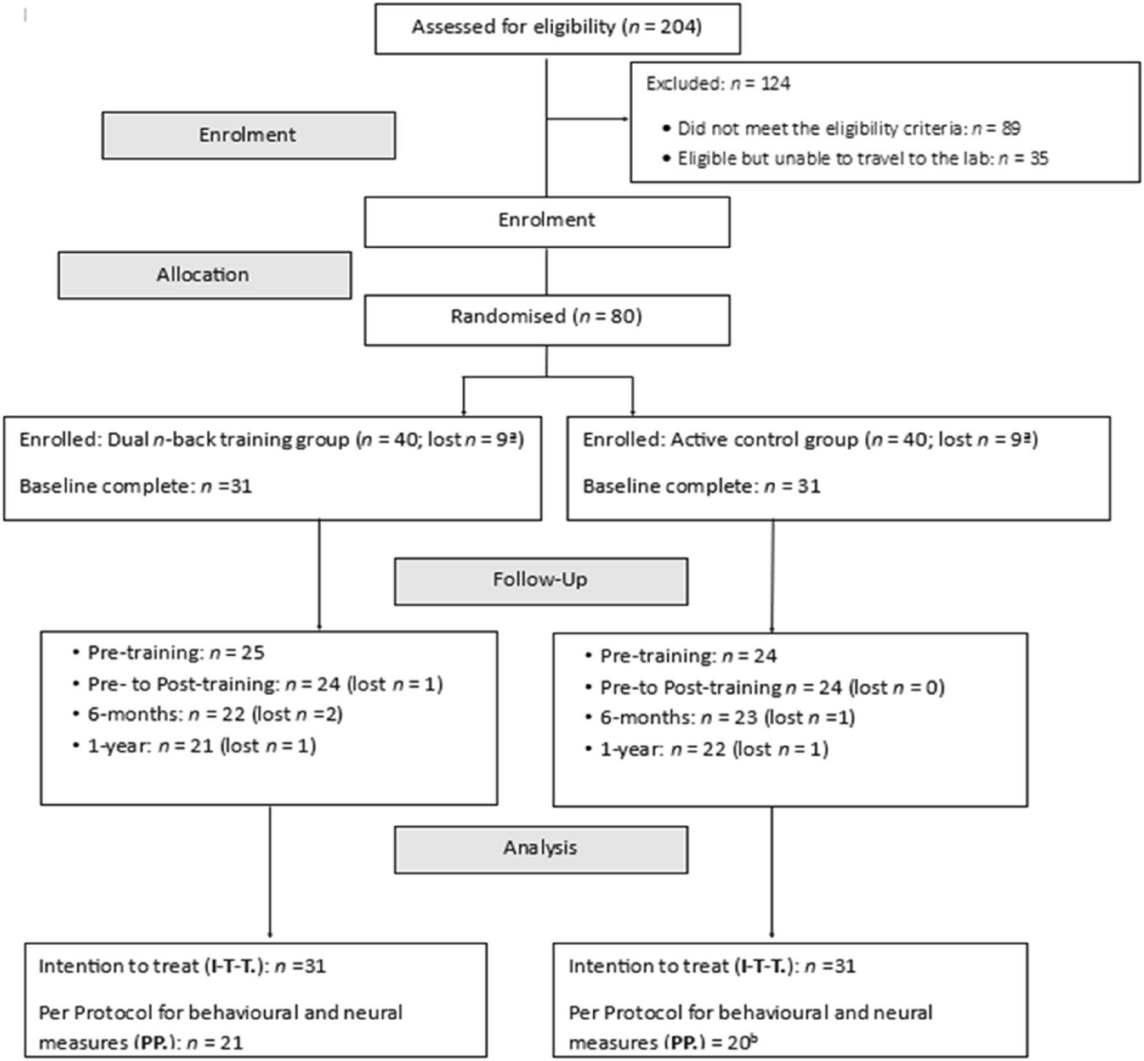
Consolidation standards of reporting trials (CONSORT) diagram of the current study including an intervention group (Dual n‐back training group) and active control group (Dual 1‐back training). Intervention group: Dual *n*‐back training. Active control group: Dual 1‐back training. ^a^See supplementary material for reasons for participants not completing baseline assessments ^b^Two participants were unable to complete behavioural and neural measures because of COVID‐19 and the subsequent closure of the lab.

Participants were allocated on a 1:1 ratio to either intervention (dual *n*‐back training) or control group using Sealed Envelope software (see Figure 1 for CONSORT diagram). After providing informed consent they completed a battery of online questionnaires followed by a lab session (approximately 2.5 h), which included assessments of WMC and EEG testing whilst completing a Flanker task. Women then independently completed 12 remote sessions of daily online training lasting approximately 30 min each over 2 weeks. Session attendance and performance were monitored daily by the experimenter. Post‐training follow‐ups were completed within 2 weeks of the final training session, at 6‐month, and at 1‐year, however, due to COVID‐19‐associated restrictions in the UK assessments of WMC and EEG testing were not completed at 6‐month or 1‐year. Women remained blind to training group allocation. On completion, women received £120.

## 3 STATISTICAL METHODS

Descriptive statistics are presented in Table 1. Chi‐square tests and independent samples bias‐corrected and accelerated (BCa) bootstrapped *t*‐tests examined group differences at baseline for the Intention‐to‐treat (ITT) sample. Bias‐corrected and accelerated 95% confidence intervals (CIs) were reported. Paired *t*‐tests (BCa bootstrapped) examined changes in working memory (as measured by the average level of ‘*n’* achieved on the dual *n*‐back task) in the intervention group from day 1 to day 12.

**TABLE 1:** Women's demographics, clinical and psychiatric history, and work characteristics at baseline.

	Intention‐to‐treat				
	Intervention group		Active control group		*p*
	*N* = *31*	(%)	*N* = *31*	(%)	
Sociodemographic					
Current age (years)	49.19 (range 34–60)		47.45 (range 36–61)		0.32
Educationᵃ					0.70
Secondary/further education	9	29.0	8	25.8	
Higher education	18	58.1	20	64.5	
History of substance misuse	1	3.2	1	3.2	1.0
Clinical—Breast cancer history					
Age at diagnosis (years)	46.9 (range 31–58)		45.0 (range 35–59)		0.29
Gradeᵇ					0.26
Grade 1	4	12.9	1	3.2	
Grade 2	9	29.0	7	22.6	
Grade 3	17	54.8	22	71.0	
Type of treatment					0.68
Chemotherapy	23	74.2	25	80.6	
Radiotherapy	27	87.1	26	83.9	
Surgery	31	100.0	31	100.0	
Time since active treatment finishedᶜ (months)	20.9 (range 6–37)		21.4 (range 6–59)		0.53
Endocrine therapy	24	77.4	21	67.7	0.39
History of psychiatric condition	9	29.0	6	19.4	0.37
Anxiety	1	3.2	0	0.0	
Depression	1	3.2	4	12.9	
Anxiety and depression	2	6.5	2	6.5	
History of a neurological condition^d^	1	3.2	1	3.2	1.0
Work					
Number of hours^e^					0.53
Full‐time	20	64.5	17	54.8	

^a^ Seven women did not disclose their highest level of education.

^b^ Two did not state the grade of their diagnosis.

^c^ One could only confirm she was between six to 60 months post‐active treatment.

^d^ One in dual n‐back training reported migraine, one in dual 1‐back training reported Essential Tremor, no other neurological conditions were reported.

^e^ One did not state the number of working hours.

Multilevel modelling (Linear Mixed Effect Models; MLMs) with autoregressive 1 compared the intervention and control group on measures of perceived cognitive abilities, workability, depressive symptoms, anxiety symptoms, and QoL over time. Fixed effects were group (intervention, control), time (baseline, post‐training, 6‐month, and 1‐year), and group × time interaction. Self‐reported data were analysed according to the intention‐to‐treat (ITT) principle. Random effects were specified as participants. A maximum likelihood method was selected for model (parameter) estimation. In line with Swainston and Derakshan,^18^ Cohen's *d* method was used to calculate the effect sizes for MLM [*d* = 2*√(F/df)]. Minimum clinical important difference (MCID) for significant changes in self‐reported symptomatology was calculated using a distribution‐based approach: MCID = $X\times SDbaseline\;[\sqrt{1‐r}$], where *X* = 1.96 and *r* = 0.2.

Mixed ANOVAs with group and time (baseline, post‐training) were conducted on outcomes on the Flanker task, neural markers of P3, ERN and Pe. When groups differed at baseline bootstrapped Univariate analysis of covariance (ANCOVAs) were performed with baseline scores as covariate.

## 4 RESULTS

### 4.1 Sample characteristics

No group differences were found for the demographic, clinical, and work‐related characteristics (all *p's* > 0.05), and similarly for those retained versus dropped out (see supplementary material Table 1).

### 4.2 Baseline characteristics

Group differences were found for PCA (BCa 95% CI [0.67, 5.60], and work mental/interpersonal demands (BCa 95% CI [−23.42, −6.08] all *ts* > 2.5, all *ps* < 0.05, all *ds* > 0.6, with the intervention group reporting worse cognitive ability, and workability than the active control group. No other differences were found (see supplementary material V. Table 2), similarly for retained versus dropped out (all *ps* > 0.05).

No group differences were found for outcomes on the Flanker task, OSpan, ERN, and Pe (see supplementary material V. table 3). Differences were found for P3 congruency and for WMC (CDT); all *ps* < 0.05.

### 4.3 Training performance

#### 4.3.1 Intervention group:

The dual *n*‐back training improved working memory from **day 1** (*M* = 1.72, *SD* = 0.40, BCa 95% CI [1.55, 1.89]) to **day 12** (*M* = 2.47, *SD* = 0.83, BCa 95% CI [2.12, 2.84]), *M* difference = 0.75, BCa 95% CI [−1.00, −0.47], *t* (24) = 5.16, *p* < 0.001, *d* = 1.03 (see Figure 4 in supplementary material VIII.). The slope of improvement was different from zero, BCa 95% CI [0.05, 0.09], *t* (24) = 7.42, *p* < 0.001, *d* = 1.48. Training compliance was 96%.

#### 4.3.2 Active control group:

The dual 1‐back group showed a consistently high level of accuracy from day 1 (*M* = 94%, *SD* = 10.56, BCa 95% CI [89.03, 97.41]) to day 12 (*M* = 96%, *SD* = 11.40, BCa 95% CI [91.56, 99.21]. Training compliance was 100%.

### 4.4 Primary outcomes

#### 4.4.1 WMC: Change detection task

**FIGURE 2:**
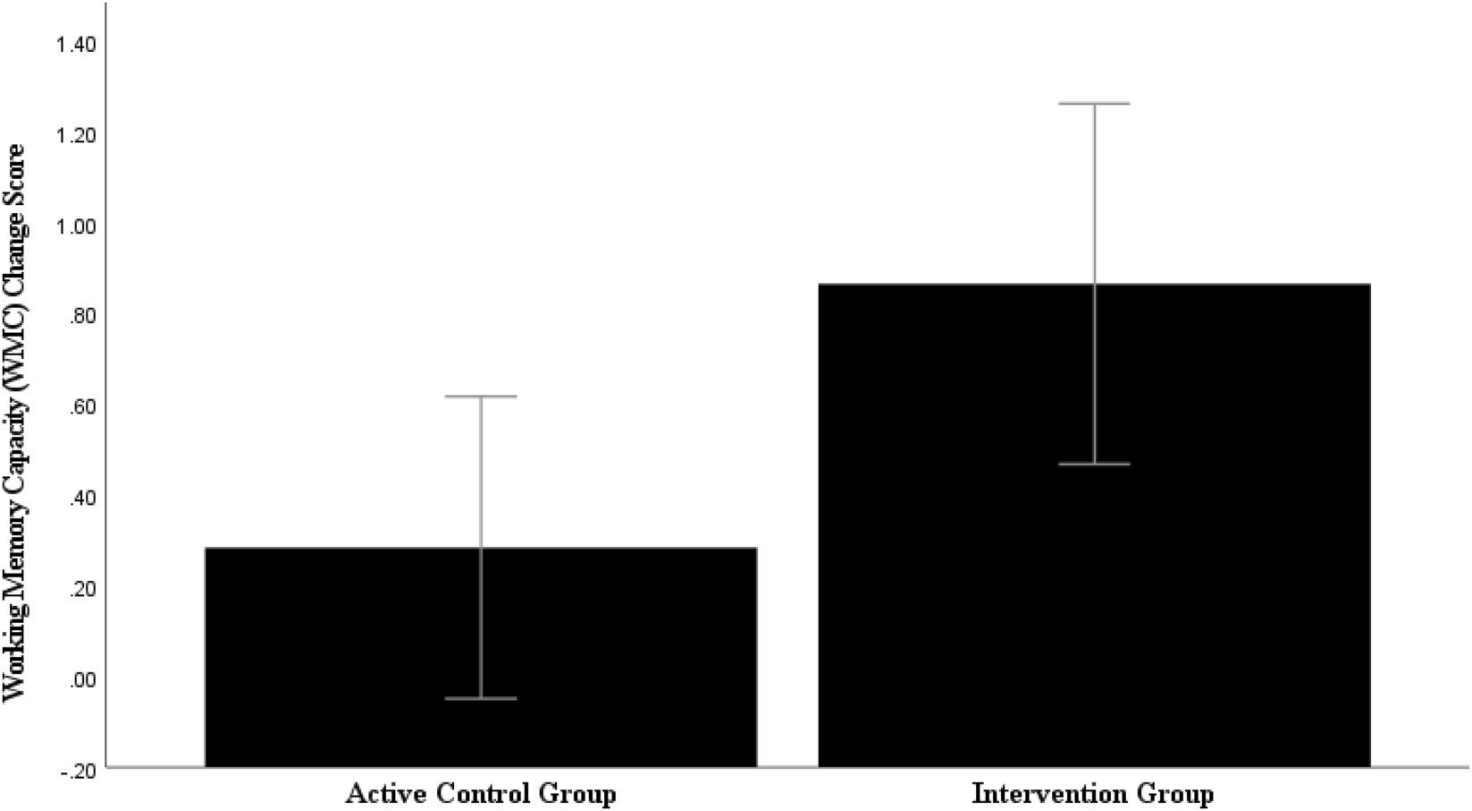
Mean working memory capacity (WMC) change scores (post—pre) on the Change detection task (CDT). Error bars = 95% CI.

The ANCOVA revealed a main effect of group, *F* (1, 38) = 5.24, *p* = 0.03, η_p_^2^ = 0.12 (Figure 2) suggesting a significant increase in WMC for the intervention group, *M* difference = 1.03, *p* < 0.001, *d* = 1.14 which was absent in the control group, *M* difference = 0.20, *p* = 0.27, *d* = 0.30 (see supplementary material VII. table 3). For *Ospan and Rumination,* no significant interactions of group × time were found (*ps* > 0.2). ***Perceived Cognitive Ability (PCA):*** The MLM interaction (group × time) was significant, *F* (3, 139.36) = 4.01, *p* < 0.01, *d* = 0.34, with the intervention group improving greater over time (*M difference* = 7.10 than the active control group (*M difference* = 5.77). The percentage of participants who met the MCID threshold for improvement was 43% (intervention group) compared with 27% (active control group).

### 4.5 Secondary outcomes

#### 4.5.1 Questionnaires

No significant interactions of time  ×  group for *Workability, Anxiety, or Quality of Life*, (all, *Fs* < 1), but a significant interaction of time × group for *depressive symptoms*, *F* (3, 130.39) = 2.93, *p* = 0.04, *d* = 0.30 was found (Intervention group: *M difference* = 8.93; Active control group: *M difference* = 4.86; see supplementary material VI. Figure 4). The percentage of participants who met the MCID threshold for improvement was 33% (intervention group) compared with 0% (active control group).

#### 4.5.2 Electrophysiological measures

##### P3 congruency on correct trials in the Flanker task

**FIGURE 3:**
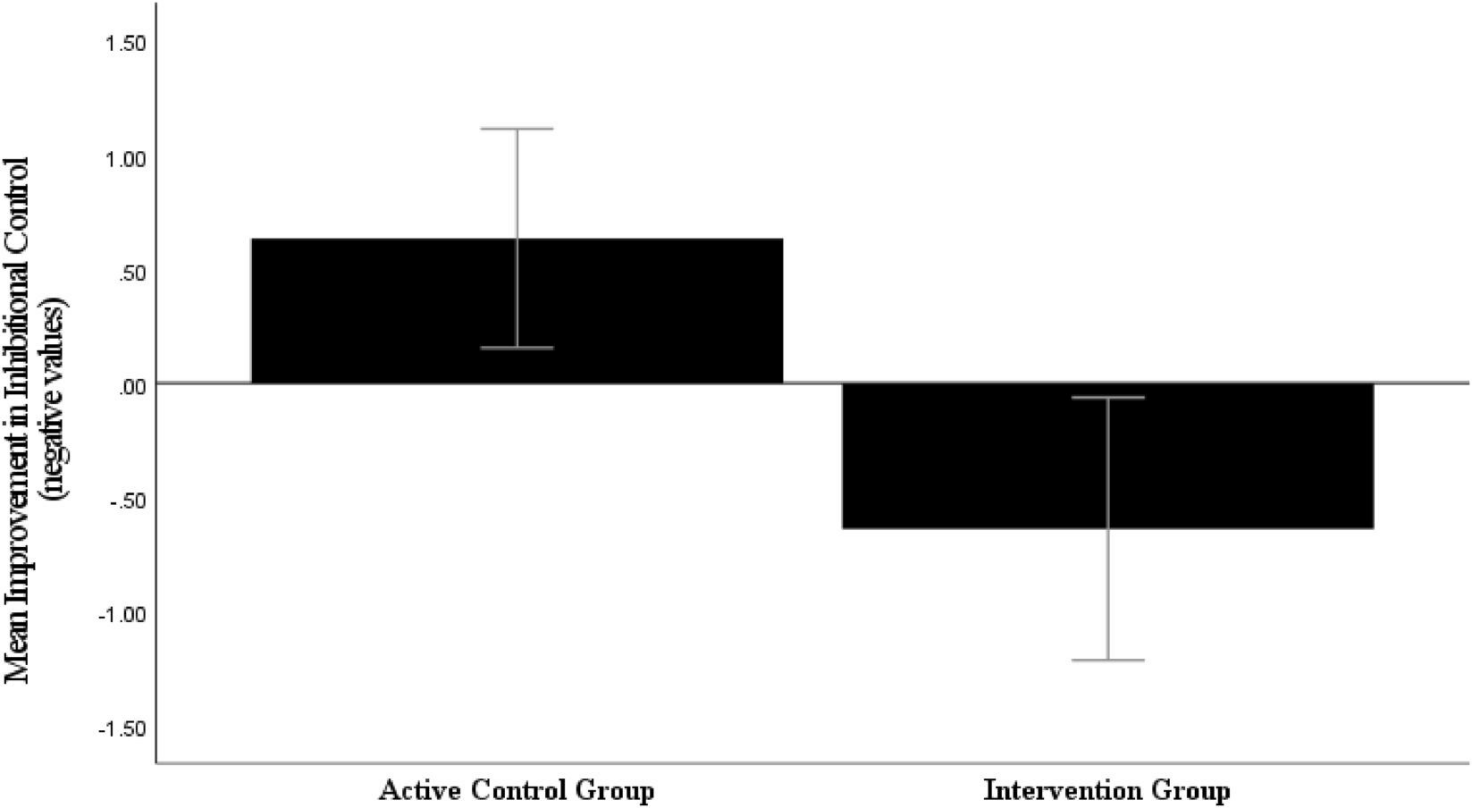
Mean P3 congruency change score (post–pre) on correct trials for both groups. Error bars = 95% CI.

Using post‐training congruency (amplitude on incongruent trials—amplitude on congruent trials) as the dependent variable, ANCOVA revealed a significant difference between the groups, *F* (1, 38) = 4.88, *p* = 0.03, η_p_^2^ = 0.11 revealing a reduction in P3 congruency in the intervention group (*M* difference = −0.64 μV, *p* = 0.02, *d* = 0.51) compared to the control group (*M* difference = 0.63 μV, *p* = 0.02, *d* = 0.62) (see Figure 3). Mixed ANOVAs showed no effects for the ERN and Pe (all *F*s < 2.14, *p*s > 0.05).

### 4.6 Further analyses

#### 4.6.1 Post error slowing on modified Flanker task

**FIGURE 4:**
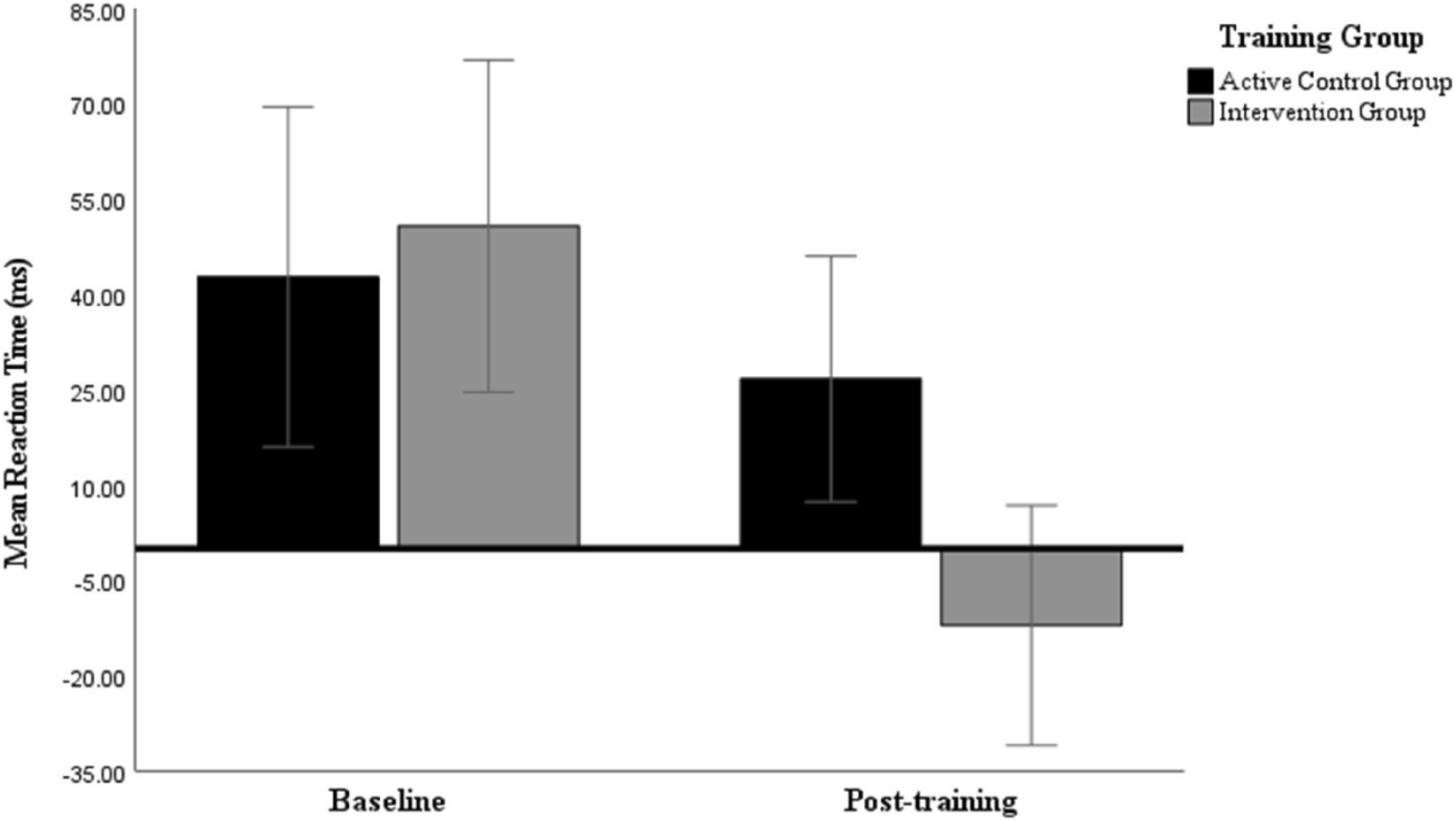
Mean post‐error slowing RT (ms) for both groups. Error bars = 95% CI.

When the difference between response reaction time on correct trials following an error (EC) and correct response (CC; EC–CC) was entered as the dependent variable main effects of time (*F* (1, 39) = 17.48, *p* < 0.001, η_p_^2^ = 0.31), and group × time interaction (*F* (1, 39) = 6.14, *p* = 0.02, η_p_^2^ = 0.14) suggested a significant reduction in post‐error slowing RT (*M* difference = 62.85 ms, *p* < 0.001, *d* = 1.00) for the intervention compared to the control group (*M* difference = 16.06 ms, *p* = 0.24, *d* = 0.28) suggesting post‐error slowing diminished in the intervention group (see Figure 4 and supplementary material VII. table 3). The groups did not differ at baseline (*p* = 0.67).

### 4.7 Exploratory analyses

Stratified analyses were conducted to see if the effects found in the analyses were subject to modulation by demographic variables. There were no effects of chemotherapy status, time since diagnosis, age at diagnosis, current age, endocrine therapy status, and education (all *ps* > 0.05).

## 5 DISCUSSION

To our knowledge, this study is the first to elucidate that adaptive dual *n*‐back training can result in transfer‐related gains to neurocognitive measures measuring WMC and processing efficiency on tasks unrelated to the training intervention, in women with a primary diagnosis of breast cancer, indicating far transfer. This supports and extends the work by Von Ah et al^11^, ^12^ that adaptive cognitive training can improve cognitive abilities in the breast cancer population longer term.

Dual *n*‐back training has improved WMC as measured by the CDT in subclinical depression.^17^ Our study is the first to show transfer benefits of the intervention on WMC to untrained tasks in women with a primary breast cancer diagnosis. Working memory supports the efficiency of a multitude of cognitive processes involved in everyday activities as well as in task‐demanding situations. The lack of an effect of training on the Ospan measure may indicate that it does not measure processes in visual working memory which are recruited by the *n*‐back task (see^33^).

The intervention group reported greater improvement in PCA and reductions in depression at 1 year follow‐up. Poorer perceived cognitive function significantly predicts higher levels of anxiety and depression, as well as worse QoL^4^ including workability in women affected by breast cancer.^34^ Our finding has important implications for women affected by cognitive impairment longer term. No significant transfer was found for workability.

Our study is the first to explore the transfer‐related gains of adaptive cognitive training on neural indices of cognitive performance in women affected by breast cancer. The intervention group showed a reduced congruency effect of the P3 amplitude suggesting successful reduction of allocation of attention to distracting information. This was accompanied by a decrease in reaction time on both congruent and incongruent correct trials indicating a greater level of cognitive efficiency. The increase in the P3 amplitude in the control group could have been driven by large baseline P3 amplitude, however, the control group did not show reduced distractibility and enhanced response inhibition post‐training. The lack of training effects on the ERN or Pe, can indicate that adaptive cognitive training may not significantly impact neural indices of error processing in this population.

Similar to Li et al.,^35^ we found that adaptive cognitive training diminished post‐error slowing which did not come at the cost of accuracy, rejecting a possibility of a speed‐accuracy trade‐off. Post‐error slowing refers to the slowing of subsequent responses following the commission of an error.^36^ According to the bottleneck error monitoring account,^37^, ^38^ error monitoring after an error requires time and engagement of central information processors, leading to a bottleneck effect (i.e., slower response) on subsequent trials. Li et al.^35^ delineate that both dual *n*‐back training (storing and manipulation of ‘*n*’ trial and current trial information) and post‐error slowing (error from previous trial and current trial information) rely on cognitive efficiency. Accordingly, *n*‐back training functions to strengthen this skill and increases cognitive efficiency, suggesting elimination of the bottleneck effect.

### 5.1 Limitations

Our study presents some limitations. Firstly, our sample size was relatively small and group differences were found on some measures at baseline. Secondly, the COVID‐19 outbreak resulted in the closure of our lab impacting some of the outcome measures. Additionally, possible COVID‐19 infections and COVID‐related cognitive problems may have influenced the findings. Thirdly, tighter eligibility criteria such as including a cognitive screener should be used in future studies to rule out other possible differences in cognition at enrolment. Fourth, future research should include a larger sample to strategically test the effects of possible confounding variables (e.g., chemotherapy status). Finally, data on menopausal status at diagnosis was not collected. Given the young age of the sample a greater decline in cognitive abilities due to treatment‐induced menopause may have been present.

### 5.2 Clinical implications

Our findings suggest that the adaptive dual n‐back training has the potential to be used by clinicians when deciding to refer patients for therapy and what type of therapy may be most helpful. This is an important consideration which can maximise treatment effectiveness. Our results provide strong justification for targeting cognitive abilities in psychological treatments as a means of empowering women with the resilience they need in everyday life.

## 6 CONCLUSION

Adaptive dual *n*‐back training can improve cognitive efficiency as measured by transfer‐related benefits on WMC, inhibitory control, perceived cognitive abilities and depression longer term, collectively indicating improved cognitive efficiency. Our intervention on improving cognitive health in a population suffering notable cognitive deficits can provide a strong base for future studies to replicate and substantiate the efficacy of this training.

## AUTHOR CONTRIBUTION

Research Question: Bethany Chapman, Elizabeth A. Grunfeld, Nazanin Derakshan; Funding: Bethany Chapman; Study Design and Analysis Plan: Bethany Chapman, Jason Moser, Elizabeth A. Grunfeld, Nazanin Derakshan; Preparation of Data: Bethany Chapman, Courtney C. Louis; Data Collection: Bethany Chapman; Analysis: Bethany Chapman, Nazanin Derakshan; Drafting initial version of manuscript: Bethany Chapman; Drafting Revisions and Final Version of Manuscript: Bethany Chapman, Nazanin Derakshan; Critical review of early versions of the manuscript: All authors.

## CONFLICT OF INTEREST STATEMENT

The authors declare that the research was conducted in the absence of any commercial or financial relationships that could be construed as a potential conflict of interest.

## ETHICS STATEMENT

Ethical approval was received from the Department of Psychological Sciences, the College Research Ethics Committee at Birkbeck College, University of London and the Economic and Social Research Council (Ref: 181,935).

## PATIENT CONSENT STATEMENT

Participants provided written informed consent online before the start of the study.

## Supporting information

Supplementary Material

## Data Availability

The dataset analysed for this study are not publicly available as the participants of this study did not consent to their data being shared.
