# Supplementary material for: Benefits of adaptive cognitive training on cognitive abilities in women treated for primary breast cancer: Findings from a 1‐year randomised control trial intervention
Source: Psychooncology. 2023 Oct 26;32(12):1848–57. doi: 10.1002/pon.6232 (PMC10946857; doi:10.1002/pon.6232)
Supplement: Supplementary file 1 — Supplementary Material [file PON-32-1848-s001.docx]

**Supplementary Material**

1. **Materials and stimuli**

***Computerised Working Memory Tasks***

The computerised tasks were carried out in a soundproof experimental booth using E-Prime 2.0 Professional Software. Tasks were shown at a distance of 70 cm on a 24-inch LED Asus VG248QE LCD computer screen with a resolution of 1920 x1080, a refresh speed of 60Hz and a response time of one millisecond.

***Change Detection Task (CDT): Working Memory Capacity***

During this task, participants were asked to remember and then compare the orientation of red rectangles (target items) shown in two sets of stimulus arrays: (1) memory array and (2) accuracy-test array. During each trial, participants were shown a white fixation cross positioned in the centre of the screen this was closely followed by the appearance of a white arrow (acting as a cue) directly above, pointing either to the left or right side of the central fixation cross for 700ms. Participants were asked to maintain their focus on the fixation cross and only attend to the rectangles on the side of the screen indicated by the direction of the white arrow. The memory array of rectangles was then shown for 100ms followed by a 900ms retention array and the accuracy-test array in which the rectangles reappeared for 2,000ms. Responses were made via the computer keyboard by pressing the ‘1’ key if there was a change in orientation of one of the red rectangles or the ‘0’ key if there was no change in the orientation of the rectangles between the (1) memory array and (2) accuracy-test array.

For each trial, one of three different possible rectangle (0.64º x 1.21º) conditions were shown including two red rectangles (2), four red rectangles (4) or two red rectangles and two blue rectangles (4D; distractors). During each stimulus array, the red (target items) or blue (distracter items) rectangles were randomly oriented in one of four positions (vertical, horizontal, left 45º, right 45º) and spaced approximately 2º apart within a 4º x 7.2º rectangular region. The two regions were centred 3º from the white central fixation cross presented on a black background.

In half of the trials, the orientation of the red rectangles in the (1) memory array matched the (2) accuracy-test array and in the other half, the orientation of one red rectangle changed between the two arrays. The rectangle condition (two red rectangles, four rectangles or two red rectangles and two blue rectangles, see figure 1 in supplementary material), orientation change (i.e., change or no change) and the direction of the arrow (right or left) were randomised and presented equally throughout the trials. WMC was calculated using the formula: K = S x (H – F) / (1 -F) [45], where S was the size of the array, H was the proportion of correct responses when the orientation of one of the red rectangles had changed (also known as the hit rate) and F was the proportion of incorrect response when the orientation of the red rectangles had not changed (also referred to as the false alarm rate). In this study, S was equal to the four red rectangles condition to avoid the ceiling or floor effects that can occur in the two-item condition and the four-item distracter condition.

**Figure 1**

*The Changed Detection Task:*


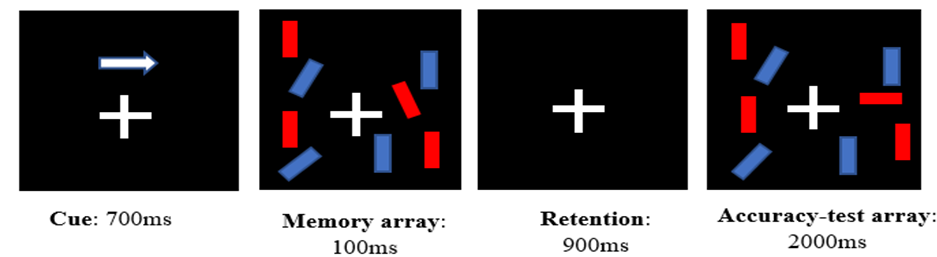


*Note.* Participants were told to remember the orientation of the red rectangles shown in the memory array and compare them to the orientation of the red rectangles shown in the accuracy-test array. Using the computer keyboard participants responded by pressing ‘1’ for change and ‘0’ for no change. In the example presented in **figure 1** participants would need to press the ‘1’ as one of the red rectangles presented on the right side has changed orientation.

***Automated Operation Span Task (OSpan): Working Memory Capacity***

The automated OSpan task a highly reliable and valid complex maths-letter span task was used. The task was composed of three practice sessions (letters only (4 trials), maths equations only (15 trials) and a combination of letter and maths equations (3 trials) and three blocks of 15 experimental trials (75 letters and 75 maths equations) each completed independently of the researcher by the participant using the computer mouse.

In the experimental trials, participants were instructed to remember a sequence of unrelated letters (‘F, H, J, K, L, N, P, Q, R, S, T and Y’; memory task component) presented in between simple maths equations (i.e., 2 + 4 =?; distractor task component). Participants were first presented with a simple maths equation, after pressing on the mouse ‘true’ and ‘false’ boxes appeared alongside a numerical answer (i.e., 6) and participants had to select the response they believed to be correct. This was followed by the presentation of a single letter. Whilst there was no overall time limit for this task, participants were only given two and a half standard deviations of their average response time (determined by the response time on the math equation practice session) to make a selection before receiving accuracy feedback and the next letter was presented. This aimed to stop the rehearsal. If no response was made within that time an error response was counted and the program moved on. The total number of letters included in each trial varied randomly from three to seven. At the end of each trial, a 4x 3 matrix of 12 possible letters was displayed. Using the mouse participants had to recall the letters presented in the correct serial order.

Feedback was provided at the end of each trial including the number of correct letters selected, number of errors made on the maths equations and overall percentage accuracy on the maths equations for the entire experiment. Participants were told that it was important to maintain an accuracy of at least 85% on the maths equations to ensure that their data could be included in the research. In addition, they were also told that to attend future experiments they must score at least 85% in the maths equations whilst maintaining high performance on the letters. The number of correct letters recalled from the three blocks of trials was summed to provide an OSpan score (also known as the partial score).

1. ***Electroencephalography***

Electroencephalography (EEG) activity was recorded continuously using BrainVision Recorder (Brain Products, Gilching, Germany) from 32, Ag-AgCl passive electrodes embedded in a standard BrainVision BrainCap (EasyCap) in accordance with the international standard 10/20 system [49] including, both left and right mastoids (TP9 and TP10) during the modified flanker task. The online reference electrode was located at FCz and the ground electrode was at AFz. Electrooculogram (EOG) activity created by vertical eye movements and blinks was recorded at FP2 and via an electrode placed approximately 1 cm below the right pupil (EOGV). Horizontal eye movement was recorded by electrodes placed on the left and right outer canthi (EOGH). Throughout data acquisition, BrainVision recorder software digitized all electrical signals at 1024 Hz and impedances were kept between 0-10kΩ. The signal was amplified with a BrainVision BrainAmp standard amplifier with a sampling rate of 1000 Hz, resolution of 0.1μV and a low cutoff of 10s (0.016 Hz) and a high cutoff of 1000 Hz. Online filters included a low cut-off filter of 0.531 Hz and a high cut-off of 70 Hz (slope for the low and high cut-off is 12 dB/octave).

Offline analyses were conducted using BrainVision Analyzer 2.2 [50]. Failed switch blocks (or failed switched mappings) (> = 60% errors) were accounted for and removed from the analysis to be consistent with behavioural analyses. Spherical splines method [51] was used for interpolation. Twenty-seven participants needed interpolation at baseline and 15 participants needed interpolation at post-training, however, none of these participants required interpolation exceeding four electrodes. Only one participant at post-training had central electrodes interpolated (Cz) that were included in the average for the ERN and CRN. Scalp electrode recordings were re-referenced to the mean of the mastoids and band-pass filtered using Butterworth zero-phase filters with a low cut-off of 0.1Hz and a high cut-off of 30 Hz (12 Db/octave roll-off). Gratton, Coles and Donchin’s method [52] was applied to correct ocular artefacts. Response-locked data (ERN, CRN, Pe) were segmented into individual epochs beginning 200ms before the response discharge and continuing for 800ms after the response production. Similarly, stimulus-locked data (P3 on congruent and incongruent correct trials) were segmented into individual epochs beginning 200ms before the stimulus onset and continuing for 800ms. A computer-based algorithm within BrainVision software was employed to detect physiological artefacts (i.e., muscle movement) and trials were rejected if the following criteria were met: (1) a voltage step exceeding 50 μV between contiguous sampling points, (2) a voltage difference of more than 200μV within a trial, or (3) a maximum voltage difference less than 0.5μV within a trial. This resulted in a loss of an average of 2.28% and 2.43% trials at baseline and 1.19% and 0.92% at post-training for response-locked data and stimulus-locked data, respectively. The remaining response-locked data were segmented into erroneous and correct responses, averaged and then baseline correction (beginning at -200.00ms and ending at 0.00ms) was applied. Separately, the stimulus-locked data were segmented into incongruent and congruent stimulus on correct trials, averaged and then baseline corrected (beginning at -200.00ms and ending at 0.00ms).

Split-half reliability was assessed using Spearman-Brown-corrected Pearson correlation coefficients between odd and even trials (*SB* = 2*r_xy_*/ (1+*r_xy_*)), **Baseline**: ERN: *r_sb_* = 0.89, *p* <.001, early pe (errors): *r_sb_* = 0.79, *p* < .001, early pe (corrects): *r_sb_* = 0.96, *p* < .001, late pe (errors): *r_sb_* = 0.70, *p* <.001, late pe (correct): *r_sb_* = 0.96, *p* <.001, P3 (incongruent trials): *r_sb_* = 0.96, *p* <.001, P3(congruent trials): *r_sb_* = 0.96, *p* <.001; **Post-training**: ERN: *r_sb_* = 0.82, *p* <.001, early pe (error): *r_sb_* = 0.72, *p* <.001, early pe (correct): *r_sb_* = 0.98, *p* <.001, late pe (error): *r_sb_* = 0.70, *p* <.001, late pe (correct): *r_sb_* = 0.96, *p* <.001, P3 (incongruent trials): *r_sb_* = 0.95, *p* <.001, P3 (congruent trials): *r_sb_* = 0.98, *p* <.001.

1. ***Intervention group: Dual n-Back Training (Adaptive cognitive training)***

**Figure 2**


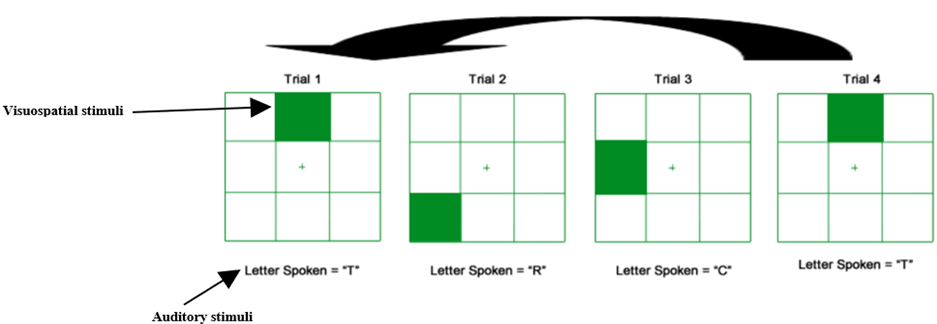
*An example of a dual 3-back training trial with a visuospatial and auditory stimuli match*

*Note.* Participants in the dual *n*-back training group were instructed to remember the position of the green box and its paired spoken consonant and respond accordingly using the computer keyboard (press ‘A’ for a visual match, ‘L’ for an auditory match and both ‘A’ and ‘L’ together for a dual match), for example, figure 2 shows a dual 3-back match as both the stimuli presented in trial 4 match what was shown 3 trials earlier.

1. ***Procedure***

Reasons for not completing baseline assessments after enrolment included the Coronavirus disease 2019 outbreak (*n* = 9), ill-health (*n* = 2) and cancer recurrence (*n* = 2). Five participants did not give a reason for their decision to withdraw before baseline (see CONSORT diagram below)

1. ***Baseline Characteristics***

**Table 1**

*Demographics, clinical and psychiatric history, and work characteristics at baseline for women retained for all three follow-ups compared to those who dropped out*

|  |  |  | | |  |  | |  |
| --- | --- | --- | --- | --- | --- | --- | --- | --- |
|  |  | **Retained** | | **Dropped out** | | |  |  |
|  |  |  | ***(%)*** |  | ***(%)*** | ***p*** | |  |
|  | ***Sociodemographic*** |  |  |  |  |  | |  |
|  | Current age (years) | 48.4 (Range 34-61) |  | 48.3 (Range 37-59) |  | .96 | |  |
|  |  |  |  |  |  |  | |  |
|  | *Education*ᵃ |  |  |  |  | .48 | |  |
|  | Secondary/further education | 14 | 33.3 | 3 | 15.0 |  | |  |
|  | Higher education | 28 | 66.7 | 10 | 50.0 |  | |  |
|  |  |  |  |  |  |  | |  |
|  | History of substance misuse | 2 | 4.8 | 0 | 0.0 | .32 | |  |
|  |  |  |  |  |  |  | |  |
|  |  |  |  |  |  |  | |  |
|  | ***Clinical - Breast cancer history*** |  |  |  |  |  | |  |
|  | Age at diagnosis (years) | 45.9 (Range 31-59) |  | 45.9 (Range 36-56) |  | .98 | |  |
|  |  |  |  |  |  |  | |  |
|  | *Grade*ᵇ |  |  |  |  | .73 | |  |
|  | Grade 1 | 3 | 7.1 | 2 | 10.0 |  | |  |
|  | Grade 2 | 10 | 23.8 | 6 | 30.0 |  | |  |
|  | Grade 3 | 28 | 66.7 | 11 | 55.0 |  | |  |
|  |  |  |  |  |  |  | |  |
|  | *Type of treatment* |  |  |  |  | .67 | |  |
|  | Chemotherapy | 34 | 81.0 | 14 | 70.0 |  | |  |
|  | Radiotherapy | 36 | 85.7 | 17 | 85.0 |  | |  |
|  | Surgical procedure | 31 | 100.0 | 31 | 100.0 |  | |  |
|  |  |  |  |  |  |  | |  |
|  | Time since active treatment finished ᶜ (months) | 20.7 (Range 6-59) |  | 22.1 (Range 6-34) |  | .93 | |  |
|  |  |  |  |  |  |  | |  |
|  |  |  |  |  |  |  | |  |
|  | Endocrine therapy | 29 | 69.0 | 16 | 80.0 | .37 | |  |
|  |  |  |  |  |  |  | |  |
|  | *History of a psychiatric condition* | 10 | 23.8 | 5 | 25.0 | .92 | |  |
|  | Anxiety | 1 | 2.4 | 0 | 0.0 |  | |  |
|  | Depression | 4 | 9.5 | 1 | 5.0 |  | |  |
|  | Anxiety and depression | 3 | 7.1 | 1 | 5.0 |  | |  |
|  |  |  |  |  |  |  | |  |
|  | *History of a neurological condition ^d^* | 1 | 2.4 | 1 | 5.0 | 1.0 | |  |
|  |  |  |  |  |  |  | |  |
|  | **Work** |  |  |  |  |  | |  |
|  | *Number of hours* |  |  |  |  | .79 | |  |
|  | Full-time | 25 | 59.5 | 12 | 60.0 |  | |  |
|  |  |  |  |  |  |  | |  |
|  |  |  |  |  |  |  | |  |

*Note.* Standard deviations are in parentheses. Intervention group: Dual *n*-back training (adaptive cognitive training). Control group: Dual 1-back training (active control training).

**Table 2**

*Means and standard deviations for perceived cognitive ability, emotional symptomology, quality of life, and workability for participants’ completion at each phase*

|  |  |  |  |  |  |  |  |  |  |  |  |  |
| --- | --- | --- | --- | --- | --- | --- | --- | --- | --- | --- | --- | --- |
|  | **Baseline** | |  | **Post-training** | |  | **6-Months** | |  | **1-Year** | |  |
|  | **Intervention group (*n* =31)** | **Active control group (*n* =31)** | ***P*** | **Intervention group (*n* =24)** | **Active control group (*n* =24)** | ***P*** | **Intervention group (*n* =21)** | **Active control group (*n* =23)** | ***P*** | **Intervention group (*n* =21)** | **Active control group (*n* =22)** | ***P*** |
| **Perceived cognitive ability (FACT-Cog)** | 11.94  (5.10) | 15.16 (5.06) | **.02*** | 13.46 (4.90) | 20.96 (6.52) | **.001*** | 18.10 (8.40) | 19.52 (6.43) | **.53** | 19.24 (7.73) | 21.14 (7.31) | **.41** |
|  |  |  |  |  |  |  |  |  |  |  |  |  |
| **Rumination (RRS)** | 53.39 (14.32) | 49.26 (12.58) | **.23** | 48.33 (12.20) | 41.67 (8.75) | **.09** | 45.57 (11.32) | 39.30 (6.46) | **.06** | 43.19 (11.15) | 41.23 (12.86) | **.57** |
|  |  |  |  |  |  |  |  |  |  |  |  |  |
| **Anxiety (HADS)** | 10.29 (5.00) | 9.23  (4.65) | **.38** | 9.92  (3.80) | 7.79  (5.26) | **.12** | 9.29  (4.78) | 6.22 (3.50) | **.02*** | 8.76  (5.11) | 6.41 (3.78) | **.09** |
|  |  |  |  |  |  |  |  |  |  |  |  |  |
| **Depression (CES-D)** | 26.48 (11.41) | 21.42 (11.37) | **.09** | 23.38 (10.75) | 13.08 (7.62) | **.001*** | 21.43 (12.03) | 11.83 (6.67) | **.002** | 18.14 (11.85) | 15.95 (12.05) | **.49** |
|  |  |  |  |  |  |  |  |  |  |  |  |  |
| **Global health status (QoL)** | 57.53 (23.80) | 66.93 (19.54) | **.09** | 64.24 (21.06) | 74.65 (17.11) | **.07** | 66.67 (17.87) | 75.72 (13.97) | **.06** | 69.44 (16.53) | 75.76 (16.04) | **.20** |
|  |  |  |  |  |  |  |  |  |  |  |  |  |
| **Workability (WLQ mental/interpersonal demands)** | 45.75 (17.03) | 33.25 (13.63) | **.002*** | 36.11 (20.44) | 25.38 (14.28) | **.04*** | 27.87 (16.07) | 19.76 (11.32) | **.06** | 22.08 (13.16) | 16.89 (4.94) | **.09** |
|  |  |  |  |  |  |  |  |  |  |  |  |  |

*Note.* Standard deviations are in parentheses. Intervention group: Dual *n*-back training group (adaptive cognitive training). Active control group: Dual 1-back training. ª Perceived cognitive ability: higher score = greater perceived cognitive ability; Rumination: higher score = greater rumination; Work mental/interpersonal demands: greater score = greater mental/interpersonal difficulty at work; Work productivity loss (%): greater score = more productivity loss; Depressive symptoms: higher score = greater level of depressive symptoms; Anxiety symptoms: higher score = greater level of anxiety symptoms; Global health status: higher scorer = better global health

**Table 3**

*Means and standard deviations for objective assessments of working memory, flanker task performance, and neural markers for Per-Protocol (PP.) sample*

|  |  |  | | |  | |  | |  | |  |  |  |
| --- | --- | --- | --- | --- | --- | --- | --- | --- | --- | --- | --- | --- | --- |
|  | | | **Baseline** | | | | |  | | **Post-Training** | | |  |
|  | | | | **Intervention group** | | **Active control group** | | ***P*** | | **Intervention group** | | **Active control group** | ***P*** |
| **Working memory capacity (CDT)** | | | 0.65  (0.96) | | | 1.33  (1.01) | | **.04*** | | 1.68  (0.70) | | 1.53  (0.90) | **.54** |
| **Ospan partial score** | | | 51.05  (18.67) | | | 52.10  (12.51) | | **.83** | | 55.24  (12.36) | | 58.90  (11.00) | **.32** |
|  | | |  | | |  | |  | |  | |  |  |
| **Flanker task:** | | |  | | |  | |  | |  | |  |  |
| **Error RT (ms)** | | | 470.59  (61.65) | | | 420.31  (85.46) | | **.04*** | | 479.84 (102.17) | | 446.13  (84.22) | **.25** |
| **Correct RT (ms)** | | | 557.71  (44.59) | | | 525.95  (58.19) | | **.06** | | 533.80 (54.61) | | 511.74  (52.84) | **.20** |
| **Post-error slowing (ms)**  **(EC - CC)** | | | 50.75  (62.62) | | | 42.75  (55.29) | | **.67** | | -12.10  (40.03) | | 26.68  (45.44) | **.01*** |
|  | | |  | | |  | |  | |  | |  |  |
|  | | |  | | |  | |  | |  | |  |  |
| **Congruent correct RT (ms)** | | | 535.37  (49.33) | | | 499.90  (56.42) | | **.15** | | 503.43 (42.61) | | 486.50 (51.98) | **.26** |
| **Incongruent correct RT (ms)** | | | 580.57  (40.59) | | | 553.49  (61.67) | | **.27** | | 554.18 (45.10) | | 537.75 (55.58) | **.30** |
| **Error correct response RT (ms)** | | | 606.19  (56.53) | | | 564.05 (93.41) | | **.09** | | 518.24 (46.85) | | 536.22 (78.50) | **.38** |
| **Correct-correct response RT (ms)** | | | 555.44  (46.55) | | | 522.30 (58.02) | | **.05** | | 530.33 (50.52) | | 509.54 (52.39) | **.20** |
|  | | |  | | |  | |  | |  | |  |  |
| **ERPs:** | | |  | | |  | |  | |  | |  |  |
| **ERN (**μV) | | | -0.20  (2.16) | | | -1.25  (1.42) | | **.12** | | -0.53  (1.32) | | -2.08 (2.55) | **.05** |
| **Early Pe (**μV) | | | 0.99  (1.84) | | | 1.64  (4.07) | | **.59** | | 0.63  (2.26) | | 1.64 (5.01) | **.50** |
| **Late Pe (**μV) | | | -0.09  (3.55) | | | 1.47  (3.59) | | **.24** | | 0.19  (3.49) | | 1.11 (3.20) | **.45** |
| **P3 on congruent correct trials (**μV) | | | 5.07  (2.00) | | | 5.81  (1.74) | | **.22** | | 6.44  (2.96) | | 6.00 (1.99) | **.58** |
| **P3 on incongruent correct trials (**μV) | | | 5.37  (2.29) | | | 5.42  (1.64) | | **.95** | | 6.11  (2.97) | | 6.24 (2.19) | **.87** |
| **P3 Congruency (incongruent -congruent trials) (**μV) | | | 0.31  (0.88) | | | -0.39  (0.84) | | **.01*** | | -0.33  (0.90) | | 0.24  (0.84) | **.04** |
|  | | |  | | |  | |  | |  | |  |  |
|  | | |  | | |  | |  | |  | |  |  |

*Note.* Standard deviations are in parentheses. Intervention group: Dual n-back training group (adaptive cognitive training). Active control group: Dual 1-back training. ᵃSeven participants were eliminated from the analysis as they did not produce enough errors (< 6) on the Flanker task; ᵇ Three participants had no error data following corrections; (ERN and Pe: Dual *n*-back training *n* =14; dual 1-back training *n* = 17)

1. ***Dual n-back Training Group Performance***

**Figure 3**

*The average level of dual n-back achieved across the 12 days of training*


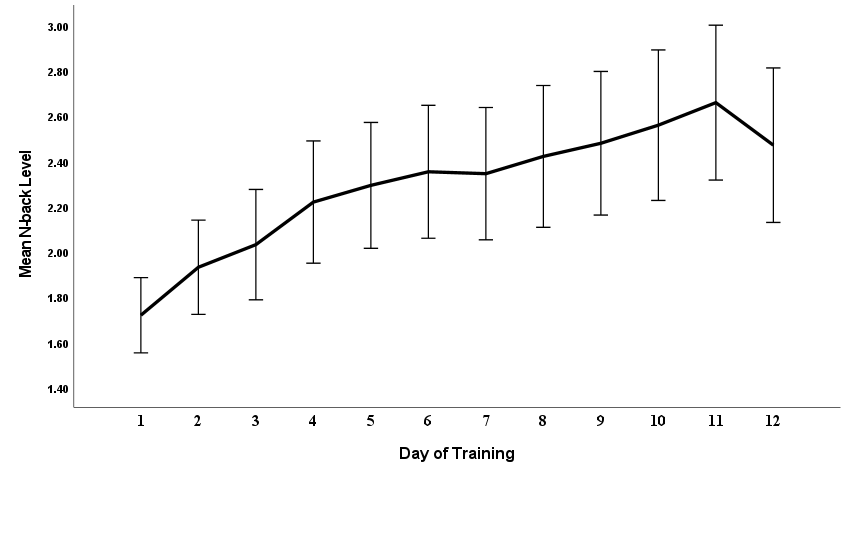


*Note***.** Error bars = CI 95%.

**VII. *Depressive symptoms***

**Figure 4**

*Mean depressive symptom scores for both groups*


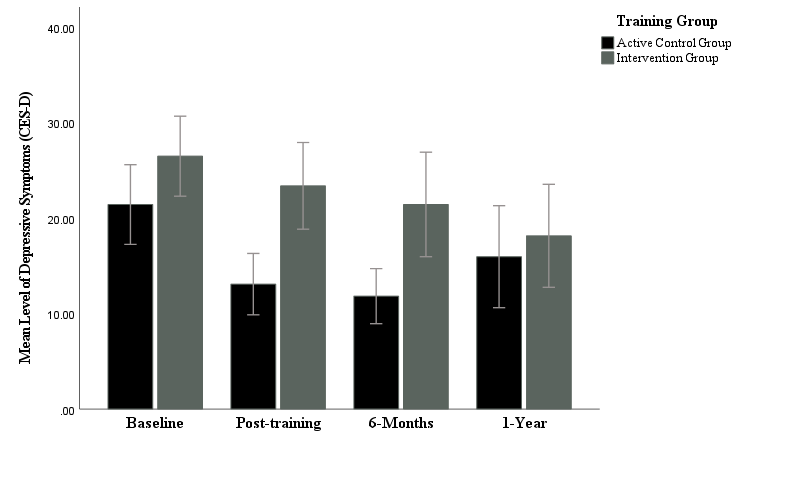


*Note***.** Error bars = 95% CI.

1. ***P3***

**Figure 5**

**
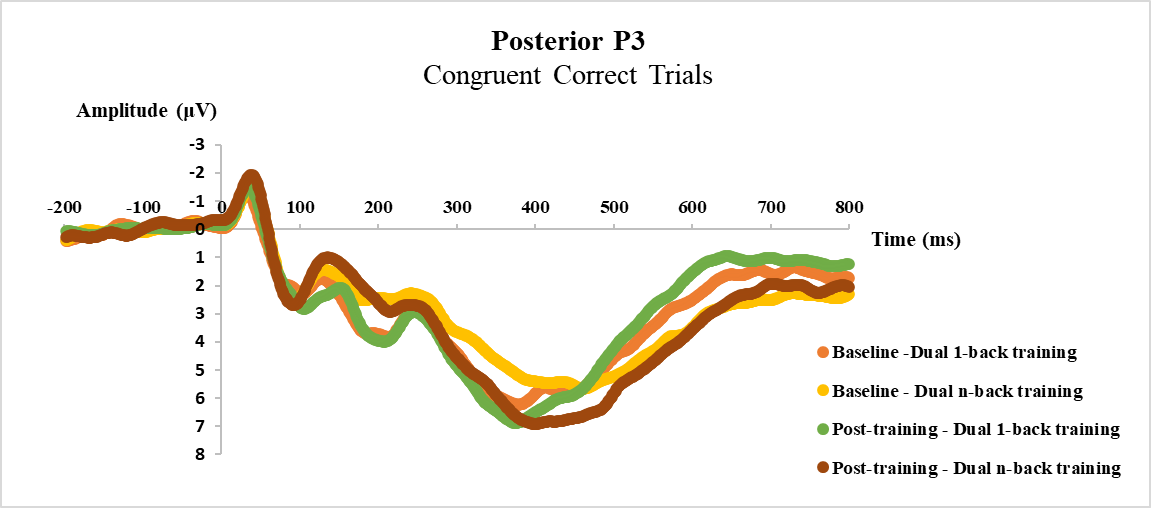
***Stimulus-locked ERP waveforms recorded from the flanker task at Pz for dual n-back and dual 1-back training*

**
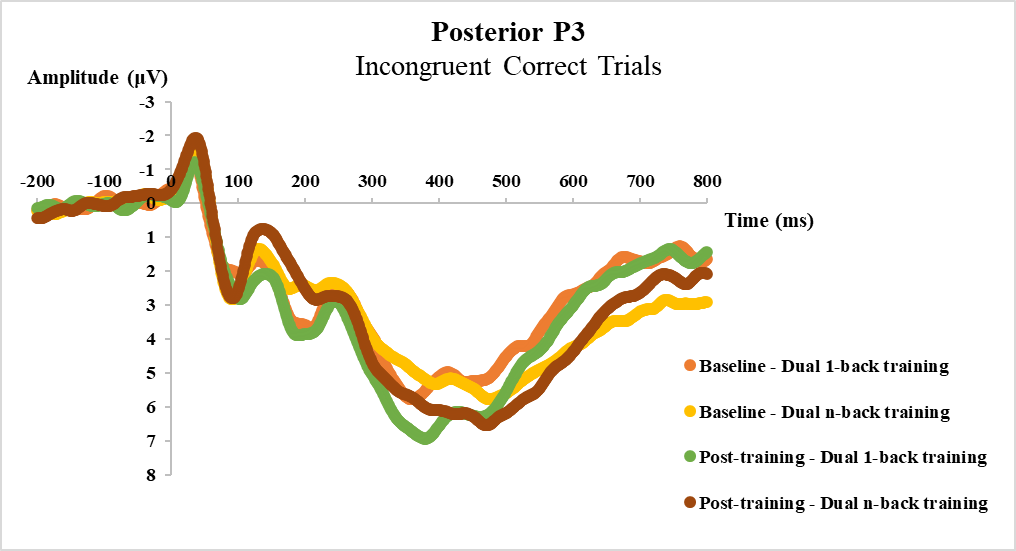
**

*Note.* In the analysis conducted above the P3 was defined as the mean activity (i.e., mean amplitude) occurring 300 to 500ms after stimulus onset.).

**XI.** ***ERN***

The number of trials included in the final analysis (based on **PP.** sample) after artefact rejection at baseline ranged from 6 to 72 for error trials (*M* =25.13, *SD* = 16.67). At post-training the number of trials included ranged from 6 to 40 for error trials (*M* = 17.00, *SD* = 8.72)^[[1]](#footnote-1)^.

**XII. *Early and late Pe***

The number of trials included in the analysis after artefact rejection at baseline ranged from 10 to 238 for congruent trials (*M* = 214, *SD* = 34.43), and ranged from 16 to 235 for incongruent trials (*M* = 205, *SD* = 32.90). At post-training, the number of trials included ranged from 175 to 239 for congruent trials (*M* = 229, *SD* = 11.78), and ranged from 166 to 238 for incongruent trials (*M* = 222, *SD* = 12.47).

1. Seven participants were eliminated from the ERN and Pe analysis as they did not produce enough error trials (< 6) on the Flanker task [↑](#footnote-ref-1)
